# In-hospital airway management training for non-anesthesiologist EMS physicians: a descriptive quality control study

**DOI:** 10.1186/s13049-017-0386-9

**Published:** 2017-04-26

**Authors:** Helmut Trimmel, Christoph Beywinkler, Sonja Hornung, Janett Kreutziger, Wolfgang G. Voelckel

**Affiliations:** 1 From the Department of Anesthesiology, Emergency and Critical Care Medicine and Karl Landsteiner Institute of Emergency Medicine, General Hospital Wiener Neustadt, Corvinusring 3-5, A 2700 Wiener Neustadt, Austria; 2 ÖAMTC Air Rescue, Vienna, Austria; 3 Department of Anesthesiology and Critical Care Medicine, Medical University, Innsbruck, Austria; 4 Norwegian Air Ambulance, Bergen, Norway; 5 Department of Anesthesiology and Critical Care Medicine, AUVA Trauma Center Salzburg, Salzburg, Austria; 6 University of Stavanger, Network for Medical Science, Stavanger, Norway

**Keywords:** Airway management, Training, Emergency physician, Emergency anesthesia, Tracheal intubation, Difficult airway algorithm, Quality control study

## Abstract

**Background:**

Pre-hospital airway management is a major challenge for emergency medical service (EMS) personnel. Despite convincing evidence that the rescuer’s qualifications determine efficacy of tracheal intubation, in-hospital airway management training is not mandatory in Austria, and often neglected. Thus we sought to prove that airway management competence of EMS physicians can be established and maintained by a tailored training program.

**Methods:**

In this descriptive quality control study we retrospectively evaluated all in- and pre-hospital airway cases managed by EMS physicians who underwent a structured in-hospital training program in anesthesia at General Hospital Wiener Neustadt. Data was obtained from electronic anesthesia and EMS documentation systems.

**Results:**

From 2006 to 2016, 32 EMS physicians with 3-year post-graduate education, but without any prior experience in anesthesia were trained. Airway management proficiency was imparted in three steps: initial training, followed by an ongoing practice schedule in the operating room (OR). Median and interquartile range of number of in-hospital tracheal intubations (TIs) vs. use of supra-glottic airway devices (SGA) were 33.5 (27.5–42.5) vs. 19.0 (15.0–27.0) during initial training; 62.0 (41.8–86.5) vs. 33.5 (18.0–54.5) during the first, and 64.0 (34.5–93.8) vs. 27 (12.5–56.0) during the second year. Pre-hospitaly, every physician performed 9.0 (5.0–14.8) TIs vs. 0.0 (0.0–0.0) SGA cases during the first, and 9.0 (7.0–13.8) TIs vs. 0.0 (0.0–0.3) SGA during the second year. Use of an SGA was mandatory when TI failed after the second attempt, thus accounting for a total of 33 cases. In 8 cases, both TI and SGA failed, but bag mask ventilation was successfully performed. No critical events related to airway management were noted and overall success rate for TI with a max of 2 attempts was 95.3%.

**Discussion:**

Number of TIs per EMS physician is low in the pre-hospital setting. A training concept that assures an additional 6﻿0+ TIs per year appears to minimize failure rates. Thus, a fixed amount of working days in anesthesia seems crucial to maintain proficiency.﻿

**Conclusions:**

In-hospital training programs are mandatory for non-anesthetist EMS physicians to gain competence in airway management and e﻿mergency anest﻿hesia.Our results might be helpful when discussing the need for regulation and financing with the authorities.

## Background

Pre-hospital airway management is a major challenge in emergency medical service (EMS) and prone to discussion and ongoing research. In a Cochrane review by Lecky et al., the skill level of the operator was judged to be key in determining efficacy of TI [1]. Although anesthesiologists are recognized specialists for airway management under difficult pre-hospital conditions [2, 3], many EMS systems must rely on physicians without appropriate training in anesthesiology or on paramedics. This is particularly true for Austria where current regulations require board certification as general practitioner or specialist in any clinical discipline, and a successfully completed 1-week course only for being licensed as an EMS physician. Clinical training related to anesthesia or intensive care is not mandatory, costly, unstructured and thus often neglected. In the German EMS system, which is more or less comparable, several major airway management disasters per year have been noted [4], thus highlighting the magnitude of the problem. In Austria, several lawsuits were conducted during the last few years dealing with unrecognized fatal esophageal intubation [5]. Additionally, there are similar cases reported in the anonymous CIRS, provided by the Austrian doctors chamber. Nevertheless, data is needed in order to convince authorities that appropriate in-hospital training is crucial for EMS personnel and subsequently patient safety. Although learning curves for TI and handling of supra-glottic airway devices (SGAs) have been identified [6, 7], case numbers necessary to gain sufficient experience in handling the entire rapid sequence induction of anesthesia (RSI) process are unknown. It is further unknown how much ongoing training (i.e. TIs or bag mask ventilation in difficult airway patients per year) is needed in order to maintain a skill level sufficient to achieve TI success rates > 95%. Data from our helicopter emergency medical service (HEMS) system strongly suggest that TI skills can not be sufficiently maintained within the service, since the necessity for advanced airway management is < 8% of all missions [8]. Furthermore, manikin training cannot replace clinical experience, and new devices such as videolaryngoscopes also require considerable experience and training; otherwise success rates might be even worse when compared with conventional laryngoscopy [9, 10]. Accordingly, we sought to evaluate the efficacy of our strict in-hospital training program assuring at least 50 TIs per year for every physician who entered EMS service as full time staff member after 3-year post-graduate training. We hypothesized that it is feasible and effective to involve EMS physicians in routine anesthesia work in the OR, and to establish adherence to our difficult airway algorithm. Thus the main purpose of our study was the proof of our EMS physician’s training concept. Since proficiency in advanced airway management is key, we sought to elaborate the pre-and in-hospital case numbers of our system. The information might be useful for the ongoing discussion about an amendment of the Austrian law defining the qualification of EMS physicians.

## Methods

General Hospital Wiener Neustadt, one of the two major teaching hospitals in Lower Austria, serves a population of approximately 110.000 inhabitants. The Department of Anesthesiology, Critical Care and Emergency Medicine is responsible for the regional ground and air rescue service responding to 3.500 emergency calls per year. Five non-anesthesiologist EMS physicians with a minimum of 3 years postgraduate experience are staffing one 24/7 ground ambulance on a rotating base under supervision of the department; air rescue is mainly staffed by consultant anesthesiologists. Besides the compulsory national EMS physician training, comprising a 1-week course, a more comprehensive departmental curriculum was established in 2000 (Table 1). During the first 3 months of EMS service, physicians work two 12 h shifts per week under direct supervision of consultants in EMS and two additional shifts in the operating room (OR) in order to acquire experience and skills. Furthermore, at least 3–5 simulator sessions together with paramedics are part of the training during the first year. After the third month, at least three 12 h in-hospital anesthesia shifts per month are mandatory. During their work in the OR, the colleagues are continuously supervised as appropriate and challenged in different settings i.e. pediatrics.

**Table 1:** Curriculum „*Anesthesia for Emergency Physicians*“

Lectures (45 min)
- Airway: anatomy and assessment
- Airway management: devices and difficult airway algorithm
- Basics of ventilation
- Pre-hospital analgesia, sedation and rapid sequence induction
- Airway management scenarios (case discussions)
In-hospital skill training (objective)
- i.v. lines / alternatives (nasal, intra-osseous)
- Induction of anesthesia (50 cases)
- Bag-mask-valve ventilation (assisted, controlled)
- Laryngeal tube, intubating laryngeal mask (20 cases, 5 in pediatrics)
- Tracheal intubation (35 cases, 10 in pediatrics - direct and video laryngoscopy)
- Use of additional devices (gum elastic bougie)
- Oral and endotracheal suction, insertion of gastric tubes
- Rapid Sequence Induction
- Knowledge and practical use of different ventilation modes
- Handling of different transport ventilators
Airway assessment (by personal mentors)
- Mini-Clinical Evaluation Exercise
- Case-Based Discussions
- Direct Observation of Procedural Skills
- Full case Simulation
Pre-hospital training
- 50 EMS missions under supervision (>10 rated NACA-grade > 3)
- Mandatory missions responding to
• Myocardial infarction
• Stroke
• Respiratory insufficiency
• Severe trauma
• Cardio-pulmonary-resuscitation
• Debriefing of training
• Allowance to do self-reliant shifts

For documentation of anesthesia, an electronic system has been established within the Department in 2005 (MCC™, Meierhofer, Munich, Germany), thus enabling an individual assessment of the number and type of anesthesia performed. In EMS, electronic documentation (NACA-X™, EDV Trimmel, Neunkirchen, Austria) has been used since 1997, enabling comprehensive analysis of all missions, interventions and procedures performed by every EMS physician.

We performed a retrospective evaluation of all in- and pre-hospital airway cases managed by every EMS physician who underwent the aforementioned training within the department during 11 years from January 2006 to December 2016. The study was performed according to the recommendations of the STROBE initiative [11]. Data obtained was handled according to current data protection guidelines. The ethical committee of Lower Austria declared the study unproblematic and granted permission (GS1-EK-4/448-2016 V). Data was analyzed employing MS Access and MS Excel (Microsoft™, Redmont, Washington, USA). Due to the descriptive nature of the study, statistical tests have not been performed.

## Results

From January 1st 2006 to December 31st 2016 32 emergency physicians with a minimum of 3 years postgraduate clinical experience underwent the aforementioned emergency anesthesia training program. Seventy-one percent proceeded their career thereafter with a residency, for the most part of them in anesthesiology (40.6%). EMS physicians were trained and supervised in 4.4% of the total anesthesia workload at General Hospital Wiener Neustadt comprising about 14.000 cases per year. Median and interquartile range of number of in-hospital TIs vs. use of SGA were 33.5 (27.5–42.5) vs. 19.0 (15.0–27.0) during initial training; 62.0 (41.8–86.5) vs. 33.5 (18.0–54.5) during the first; and 64.0 (34.5–93.8) vs. 27 (12.5–56.0) during the second year.

**Fig. 1:**
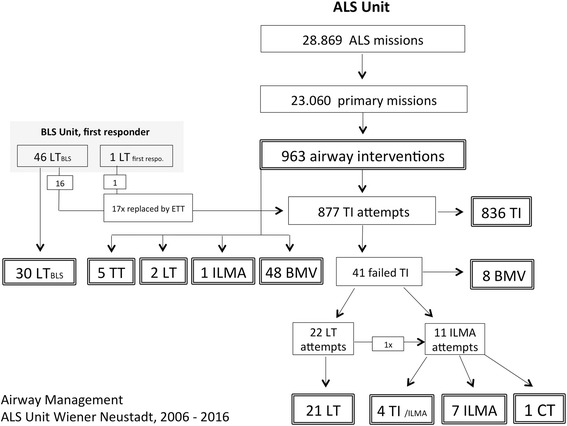
Airway Management ALS Unit Wiener Neustadt. This figure includes all airway interventions performed in context with primary missions of the ALS Unit Wiener Neustadt from 2006 to 2016

During the same 11 years observation period, EMS physicians responded to 23.060 primary missions and 5.809 aborted missions or inter-hospital transfers. In 963 of all primary missions (4.2%) an airway intervention was required, at least transiently. Figure 1 depicts all airway interventions documented in the entire study period.

TI was indicated in 877 patients, due to the indications outlined in Table 2. Rapid sequence induction was required in 313 cases (35.7%). The overall TI success rate was 95.3%. Based on the institutional difficult airway algorithm, an SGA was established after two attempts of failed direct laryngoscopy intubation in 33 patients in cardiac arrest (*n* = 22) or other critical conditions (*n* = 11). 15 of the SGA patients (all in cardiac arrest at EMS arrival) died on scene, but 18 patients were successfully oxygenated, ventilated and subsequently transported to hospital.

**Table 2:** Indications for tracheal intubation

Indication for ETI	*n*	%
CPR	564	64.3
Trauma	88	10.0
Other	72	8.2
Intracranial bleeding / stroke	67	7.6
Unconsciousness	35	4.0
Respiratory insufficiency	21	2.4
Cardiac failure	16	1.8
Suspected intoxication	14	1.6
	877	100.0

Only 8 patients were transported with ongoing bag mask valve ventilation (BMV) due to a cannot intubate scenario (*n* = 4), extreme short transport times to the hospital (*n* = 3) and one during established CPR in an ambulance vehicle. 2/8 patients improved during transport; 4/8 needed intubation immediately after admission, and 4/8 survived to hospital discharge. In 17 patients who had to be managed with an SGA or BMV after failed tracheal intubation, oxygenation at arrival was sufficient in all but one case, whereas normo-ventilation was achieved only in 3 patients. Mortality rate of these patients was 53% due to the criticality and underlying diagnosis. During the entire study period only one surgical airway attempt in a cardiac arrest patient was reported.

In 7 patients the emergency physicians abandoned TI in favor of BMV (*n* = 4) or SGA (*n* = 3) due to an expected difficult airway. In 17 cases, the SGA established by BLS units was changed to TI by emergency physicians. These were performed after 2013, based on the recommendation to replace an SGA as soon as possible in order to avoid severe complications [12].

## Discussion

The impact of emergency airway management in pre-hospital patients on outcome is, at least in part, determined by the operator’s proficiency [1, 3, 13, 14]. Given the fact that the absolute number of patients requiring intubation is low [15, 16], airway management proficiency and intubation skills are not likely to be generated or maintained in the pre-hospital field [8]. Taken together, an airway intervention was required in 87.5 missions per year or in 1/30 missions. Thus, there is only one airway intervention every 4.2 days or, due to 12 working hour restriction, every 8.3th shift. Accordingly, the number (median and interquartile range) of TI vs. SGA cases per year per EMS physicians is very low 9.0 (5.0–14.8) vs. 0.0 (0.0–0.3). The total number of 36 SGA cases during the 11 years observation period outline how seldom this device must be employed, namely 3.3 times per year, every 640 missions, or every 114 days. In pediatric patients, only one use of an SGA was documented in a cardiac arrest setting during the 11 years. These low numbers are due to a significant over-triage of the physician EMS unit, which is specific for the dispatch policy in Lower Austria employing a priority dispatch program. In this regard it is further noteworthy that up to 90% of all BLS ambulances within the region are staffed with voluntary EMS technicians or paramedics with less competence when compared with other countries. Thus less than 25% of all physician EMS unit missions are referring to life-threatening situations [17].

### Clinical training and performance

Despite the fact, that learning curves for TI or insertion of SGAs have been assessed [18–22], little is known about the efficacy of an initial and ongoing airway management program for EMS physicians. Results from our study demonstrate that a structured teaching and consequent on-going in-hospital training program enables even non-anesthetist EMS physicians to successfully manage patients airways. Significant experience (62 TIs vs. 33 SGAs) was achieved within the first year, and maintained during the second year (64 TIs vs. 27 SGAs). As mentioned, TI numbers in the pre-hospital service were low and rarely exceeded 10 airway interventions per physician per year in our ground EMS. Nevertheless, 95.3% were successfully intubated within 2 attempts. Strict adherence to the airway algorithm required use of an SGA or bag-mask ventilation when the second TI attempt failed, or when the airway was judged difficult. With this approach, the remaining 4.7% were successfully oxygenated and ventilated. There were no severe adverse events like “cannot intubate—cannot oxygenate” situations reported during the entire study period.

There is no doubt that proficiency and skills are crucial for all personnel in EMS care and several publications shed light on this issue [23, 24]. In this regard, learning curves have been established. For a 90% TI success rate numbers between 47 and 80 intubations have been postulated [6, 7, 20, 25]. Moreover, airway management skills have been shown to decline [26] early after initial training. For the SGA, at least 40 insertions in patients are recommended to achieve a first or second attempt success rate of 86 or 96%, respectively [21]. In this regard, our findings strongly support the hypothesis that a focused initial airway management instruction and continuous training in the OR to the described extent will result in a > 95% TI success rate, even by prior completely inexperienced EMS providers.

### Comprehensive training concept

However, competence in airway management comprises more than the manual skill of advancing the tube into the trachea: as depicted in Table 1, initial training imparts techniques of airway assessment as well as different modes of RSI (according to our standard operating procedure), appropriate use of medication, optimized setting of the ventilator and so forth. Furthermore, trainees in our anesthesia department should become acquainted to the following aspects of airway management. First, the indication for induction of anesthesia and TI must be made deliberately. In pre-hospital intubated trauma patients who would not have required TI, almost all outcome parameters were significantly worse when compared to spontaneously breathing controls [27]. Second, strict adherence to a pre-oxygenation protocol and difficult airway algorithm will further increase overall airway management success and patients outcome [28, 29]. Third, competence in BMV is crucial and finally enables survival in scenarios were an airway cannot be established [30, 31]. Forth, when induction of emergency anesthesia is necessary, emphasis must be given to cardiovascular side effects of anesthetic drugs. In this regard hypotension after induction of anesthesia has been identified as an adverse outcome indicator [32]. Fifth, after successful intubation hypo- and hyperventilation must strictly be avoided since failures in ventilation negatively affect outcome [33]. Taken together, airway management is complex and may not be reduced to a single intervention such as TI [34]. This might explain why physicians might have significantly fewer pre-hospital TI failures when compared with non-physicians [3]. Fullerton et al. even raised the question whether non-anesthesiologists should perform pre-hospital RSI. The authors observed a higher TI failure rate during RSI and speculated that this was caused by a lack of training opportunities and infrequency of clinical experience [2]. Our ground-based EMS system is predominantly staffed with non-anesthesiologist physicians with the Austrian legal requirement for professional medical license of at least three years of post-graduate clinical experience. Nevertheless, a well-defined training catalogue in combination with a difficult airway algorithm enabled a TI success rate of 95%, and equally important, successful management of patients with failed TI attempts. This sheds further light on the necessity to train alternative airway devices such as the laryngeal mask or laryngeal tube in-hospital, because use of the SGAs are extremely rare in the pre-hospital setting occurring in less than 1 of 640 missions. It is further noteworthy, that in critical patients with failed TI, oxygenation could be restored in 14/15 patients with use of SGAs and BMV, but hypercapnia was observed after admission in 14/17 patients. Thus, tight control of end-tidal carbon dioxide levels is crucial, but operators must be aware that there is a significant and variable risk of mismatch between EtCO_2_ and PaCO_2_ measurements [35, 36].

### Limitations of the study

The study was designed as a descriptive quality control study of our airway management training concept and subsequently, airway management performance of our non-anesthesiologist EMS physicians. Accordingly, our data lacks information about the overall outcome of patients subjected to TI, SGA or BMV management. We are further unable to report on the quality of the airway procedure i.e. times until the airway was secured, declines in oxygen saturation or blood pressure. Nevertheless, we demonstrated that non-anesthesiologist EMS physicians are able to perform well in the pre-hospital setting when appropriate in-hospital training is granted.

## Conclusion

Our data confirm that the number of TIs per EMS physician is low in the pre-hospital setting. Accordingly, a fixed amount of working days in anesthesiology is necessary in order to maintain proficiency. An established training concept that assures an additional 60 + TIs per year appears to be feasible and minimize failure rates. Our results might be helpful to discuss with authorities the need for regulation and financing of EMS.

## Abbreviations

ALS: Advanced life support
BLS: Basic Life Support
BMV: Bag-valve-mask ventilation
CT: Cricothyrotomy
ETT: Endotracheal tube
ILMA: Intubating laryngeal mask
LT: Laryngeal tube
TI: Tracheal intubation
TT: Tracheostomy
